# Draft Genome Sequence of *Bacillus subtilis* ALBA01, a Strain with Antagonistic Activity against the Soilborne Fungal Pathogen of Onion *Setophoma terrestris*

**DOI:** 10.1128/genomeA.00455-16

**Published:** 2016-06-02

**Authors:** Andrea G. Albarracín Orio, Romina A. Tobares, Daniel A. Ducasse, Andrea M. Smania

**Affiliations:** a Laboratorio de Biología Molecular, Facultad de Ciencias Agropecuarias, Universidad Católica de Córdoba, Córdoba, Argentina; b Centro de Investigaciones en Química Biológica de Córdoba, CIQUIBIC, CONICET, Departamento de Química Biológica, Facultad de Ciencias Químicas, Universidad Nacional de Córdoba, Ciudad Universitaria, Córdoba, Argentina; c Instituto de Patología Vegetal IPAVE CIAP-INTA, Córdoba, Argentina

## Abstract

*Bacillus subtilis* is a nonpathogenic bacterium that lives in soil and has long been used as biological control agent in agriculture. Here, we report the genome sequence of a *B. subtilis* strain isolated from rhizosphere of onion that shows strong biological activity against the soilborne fungal pathogen *Setophoma terrestris*.

## GENOME ANNOUNCEMENT

Biocontrol is a reliable alternative to chemical fungicides, which have raised serious concerns regarding food contamination and environmental pollution. Biocontrol is eco-friendly, safe, and may provide long-term protection to a crop (1). Plant growth-promoting rhizobacteria (PGPR) are naturally occurring soil microorganisms that colonize plant roots and benefit plants by increasing growth and/or reducing disease impact. Several *Bacillus* spp. representing typical PGPR have been widely studied and applied as efficient and stable biocontrol agents due to their ability to form heat- and desiccation-resistant spores (2). Bacteria belonging to the genus *Bacillus* are considered to be safe microorganisms that hold the remarkable ability to synthesize a vast array of beneficial substances for agronomical and industrial purposes (3). We have isolated a strain of *Bacillus subtilis* subsp. *subtilis* from the rhizosphere of onion plants (Bahía Blanca, Argentina) that was capable of strongly inhibiting the growth of *Setophoma terrestris* in vitro. We named it *B. subtilis* strain ALBA01. Interestingly, we observed a high growth inhibition of *S. terrestris* on plates containing cell-free supernatant of *B. subtilis* ALBA01 previously grown in the presence of the fungus (4). No antagonistic activity against two other onion pathogens, *Fusarium oxysporum* f. sp. *cepae* and *Fusarium proliferatum*, was observed. To gain insight into the features of *B. subtilis* ALBA01, we sequenced and annotated its genome sequence. Whole-genome sequencing was performed using a paired-end (PE) 2 × 100-bp library on an Illumina HiSeq 1500 (INDEAR Genome Sequencing facility, Argentina). The A5 pipeline (5) was used to execute *de novo* assembly. Reads were assembled into 28 scaffolds, with an average scaffold size of 147,128 bp. Annotation of the genome was done by uploading the scaffolds to the Rapid Annotations using Subsystems Technology (RAST) server (6) and by using the SEED-based method on this server. The genome sequence of *B. subtilis* ALBA01 is composed of 4,119,571 bp, with a mean G+C content of 44%. The annotation revealed 4,303 open reading frames (ORFs). Three families of *Bacillus* lipopeptides (surfactins, iturins, and fengycins) have mostly been studied for their antagonistic activity against a wide range of potential phytopathogens, including fungi (7). Then, we decided to analyze the genome for the presence of genes encoding antimicrobial peptide synthetases. Four ORFs showed high identity levels to those from the *srfA* locus required for the production of the lipopeptide antibiotic surfactin. We also found an ORF with 98.77% identity to *pksD*, a gene encoding an enzyme involved in polyketide synthesis, and another with 80.66% identity to the fengycin synthetase gene, *fenB*. No coding sequences were found to be related to *ituD* (iturin A synthetase D), *bamA* (bacillomycin D synthetase A), *bmyA* (bacillomycin L. synthetase A), and *mycA* (mycosubtilin synthase subunit A).

### Nucleotide sequence accession numbers.

This whole-genome shotgun project has been deposited at DDBJ/ENA/GenBank under the accession no. LVYH00000000. The version described in this paper is version LVYH01000000.
